# Beauty of the beast: anticholinergic tropane alkaloids in therapeutics

**DOI:** 10.1007/s13659-022-00357-w

**Published:** 2022-09-16

**Authors:** Kyu Hwan Shim, Min Ju Kang, Niti Sharma, Seong Soo A. An

**Affiliations:** 1 Bionano Research Institute, Gachon University, 1342 Seongnam-daero, Sujeong-Gu, Seongnam, 461-701 South Korea; 2 Department of Neurology, Veterans Health Service Medical Center, Veterans Medical Research Institute, Seoul, South Korea

**Keywords:** Tropane alkaloids, Poisonous plants, Anticholinergic action, Muscarinic and nicotinic receptors, Therapeutics, Anticholinergic burden

## Abstract

Tropane alkaloids (TAs) are among the most valued chemical compounds known since pre-historic times. Poisonous plants from Solanaceae family (*Hyoscyamus niger, Datura, Atropa belladonna*, *Scopolia lurida, Mandragora officinarum, Duboisia*) and Erythroxylaceae (*Erythroxylum coca*) are rich sources of tropane alkaloids. These compounds possess the anticholinergic properties as they could block the neurotransmitter acetylcholine action in the central and peripheral nervous system by binding at either muscarinic and/or nicotinic receptors. Hence, they are of great clinical importance and are used as antiemetics, anesthetics, antispasmodics, bronchodilator and mydriatics. They also serve as the lead compounds to generate more effective drugs. Due to the important pharmacological action they are listed in the WHO list of essential medicines and are available in market with FDA approval. However, being anticholinergic in action, TA medication are under the suspicion of causing dementia and cognitive decline like other medications with anticholinergic action, interestingly which is incorrect. There are published reviews on chemistry, biosynthesis, pharmacology, safety concerns, biotechnological aspects of TAs but the detailed information on anticholinergic mechanism of action, clinical pharmacology, FDA approval and anticholinergic burden is lacking. Hence the present review tries to fill this lacuna by critically summarizing and discussing the above mentioned aspects.

**Graphical Abstract:**

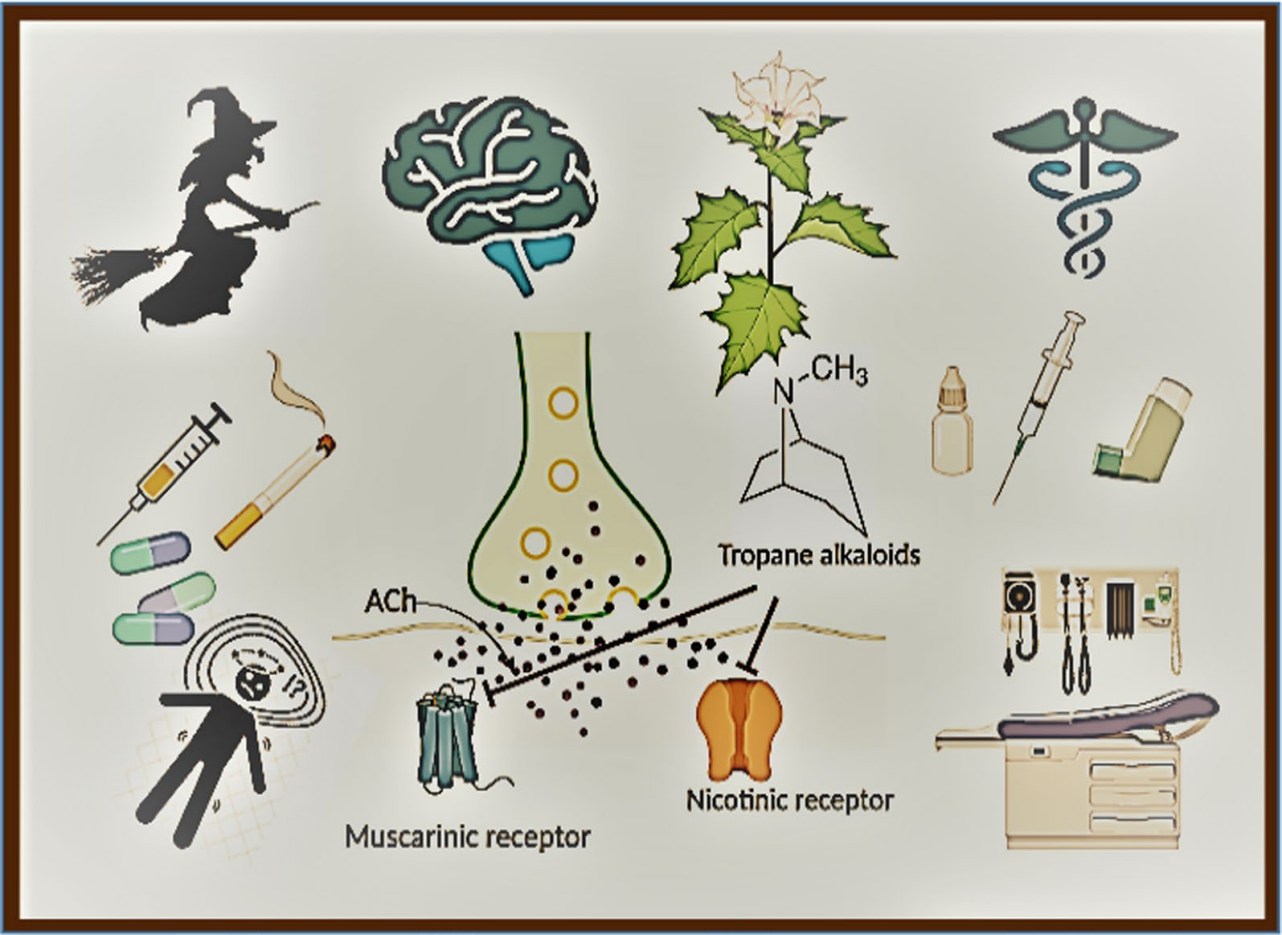

## Introduction

Tropane alkaloids (TAs) belonged to a class of about 200 alkaloids with a distinctive bicyclic tropane ring in their chemical structure [1]. Occurrences and distributions of tropane alkaloids are reported in the plants from families like, Convolvulaceae, Erythroxylaceae, Solanaceae, Proteaceae, Euphorbiaceae, Rhizophoraceae, and Cruciferae [2]. Interestingly, the highest concentrations of TAs are present in Solanaceae (*Hyoscyamus niger, Datura, Atropa belladonna*, *Scopolia lurida, Mandragora officinarum, Duboisia*) and Erythroxylaceae (*Erythroxylum coca*) [3, 4]. The existing important bioactive compounds in these plants are atropine, hyoscyamine and hyoscine (scopolamine). These plants are poisonous upon ingestion and can have dire consequences. The high doses of their extracts and/or compounds could result in delirium, stupefaction and intense hallucinations [5]. The lethal dose of atropine is around 10 mg while that of scopolamine is much lower (2–4 mg) [6] Relatively very high concentration of atropine (0.1 mg/ seed) and scopolamine (3.85 mg/g leaves) is present in *Datura stramonium* (Jimsonweed) and *Datura inoxia* (Moon flower), respectively [6]. The scopolamine content of *Scopolia lurida* (1.5 mg/100 mg dry weight of leaves) is reported to be higher than that of *A. belladonna* [4, 7]. Scopolamine is listed among the indispensable medicines of the world by the World Health Organization (WHO) [8]. Both *Atropa* and *Datura* species accounts for roughly 0.2–0.8% of total alkaloids with a fairly low scopolamine (hyoscine) content. So they are not commercially used for the extraction of TAs. To meet the needs of pharmaceutical industry, another plant (*Duboisia*) is cultivated on a commercial scale for being a rich source (2–4%) of TAs (constituting about 60% scopolamine, and 30% hyoscyamine). The pharmaceutical industry need uninterrupted high supply of TAs (specially, scopolamine and atropine) which is a limiting factor in the field grown plants as various biotic and abiotic factors affect the production and concentration of TAs. Hence, to overcome this problem, unconventional methods, like metabolic engineering is used to increase the availability [9–12].

TAs are the secondary plant metabolites which have been used since ancient times in traditional medicine [13], poison [14–16], cosmetics [17], recreational purposes [18, 19] and, blood sport [16, 20]. The leaves of *Erythroxylum coca* (source of cocaine) were chewed to increase the stamina and are also consumed during religious sacrifices [21]. Deliriant poisoning was prevalent among American youth in 1960s by deliberate intake of ‘*Asthmador*’ (asthma medication containing extract of *D. stramonium*)*,* for experiencing euphoria [22]. In fact, due to hallucinogenic and psychoactive properties TAs are associated with the magic/rituals by many ethnic groups all around the world. Under the influence of smoke from these plants, the priests were reported to deliver divinations after entering a trance [23]. In ancient times “flying salves” were made from these plant extracts (*Atropa belladonna, Hyoscymaus niger* and *Mandragora officinarum*) which when applied to the broom stick get absorbed through the skin of witches and imparted the power of flying to them [24–26] as mentioned in the ancient texts.

**Fig. 1:**
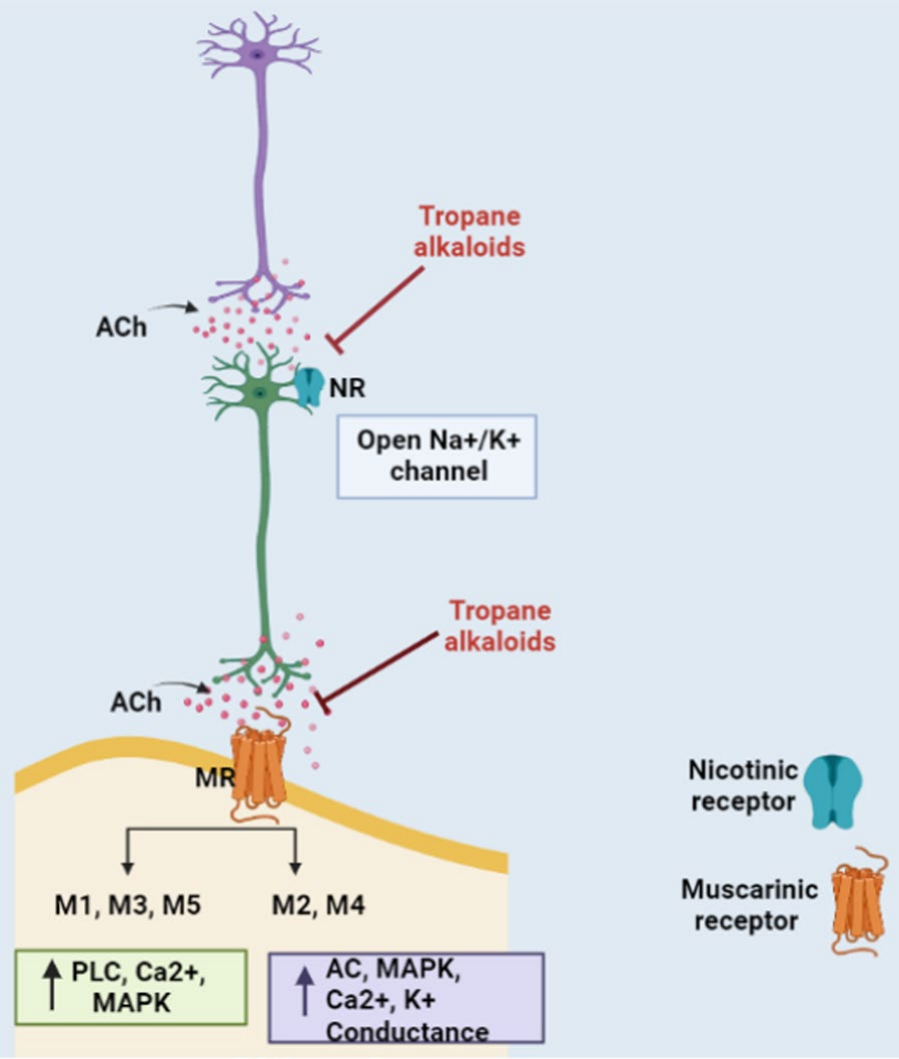
Anticholinergic Action of Tropane Alkaloids. Tropane alkaloids are anticholinergic in action. TAs competitively bind to muscarinic and/or nicotinic receptors and block ACh transmission. MR: Muscarinic receptor; NR: Nicotinic receptor; ACh: Acetylcholine; PLC: Phospholipase C; AC: Adenylyl cyclase; MAPK: Mitogen-activated protein kinase [Prepared using Biorender.com]

TAs display anticholinergic effect as they block neurotransmitter acetylcholine (ACh) action in the central and peripheral nervous system (CNS and PNS) by binding at either muscarinic receptors (mAChR) or nicotinic acetylcholine receptors (nAChR), to a lesser extent (Fig. 1).

As a consequence of this anticholinergic action both CNS and PNS are affected and the symptoms are manifested by changes in heart rate & respiration, muscle contraction, hallucination etc. [27]. Even though all the TAs share structural similarity due to the presence of tropane ring in the scaffold, they exhibit considerably different pharmacological effects. Atropine, hyoscyamine, scopolamine and cocaine can cross the blood–brain barrier (BBB) to effect the CNS [28, 29], while the calystegine cannot, owing to its hydrophilic nature. As a result, calystegines are unable to show psychoactive results [3]. Cocaine blocks the reuptake of neurotransmitters like dopamine, noradrenaline and serotonin in the synaptic cleft [30]. As compared to atropine, scopolamine is more suitable in situations where decreased parasympathetic activity is required. In PNS it relaxes smooth muscles and decrease the body secretions. Additionally, in CNS scopolamine causes drowsiness, unlike atropine [31].

Hyoscine, the first drug to be called “truth serum” (a drug used to extract truth from the criminals), was accidently discovered by Dr. Robert House in early twentieth century [32]. Over twenty active pharmaceutical ingredients (API) having tropane moiety are being manufactured by various pharma companies [33]. The natural and synthetic TAs are anti-secretory [34] anti-spasmodic [35], antiemetic in action and are being used for treating allergies, asthma and chronic obstructive pulmonary disease (COPD) [36], urinary dysfunction, irritable bowel syndrome (IBS) [35], post-operative nausea and vomiting (PONV) [31], local anaesthetics [37], ophthalmological eye drops [38], antidote against organophosphorus compounds poisoning [39].

The common side effects of TAs are related to parasympathetic stimulation; decreased body secretion resulting in dry mouth and eyes, constipation, urine retention, tachycardia, depressive activity in CNS and delirium [38]. Due to the side effects caused by TA medications, their prescription may be considered unsuitable in some situations. Still, for some critical clinical conditions, like death rattle [40] the benefits of using TAs overrides their side effects.

The available reviews focused on TA occurrence and distribution [41], pharmacological outline [3, 42], biosynthesis [3, 43], biotechnology [44], poisoning [45–47], production techniques [48] and cytotoxicity [49]. However, the detailed information on anticholinergic mechanism of action, clinical pharmacology and anticholinergic burden is lacking. Therefore, in an effort to search the benefits of these so called venomous or dark compounds, a comprehensive information on therapeutic action mechanism and clinical pharmacology was collected using PubMed, Google Scholar, Scopus as online databases until May, 2022 with key words being tropane alkaloids, anticholinergic, clinical pharmacology, anticholinergic burden, therapeutics, FDA approval and toxicity. We included studies conducted on human and animals for clinical pharmacology. Additional information was extracted from the citations mentioned in the articles. However, the observational studies and any other unrelated pharmacological activity on the topic were excluded. Hence, this review not only summarized the literature but also discussed it critically. This information will be helpful in better understanding of the role of TAs in therapeutics and designing new pharmacophore with better therapeutic profile hence will contribute to new vistas for advanced research on TAs.

## Tropane alkaloids

**Fig. 2:**
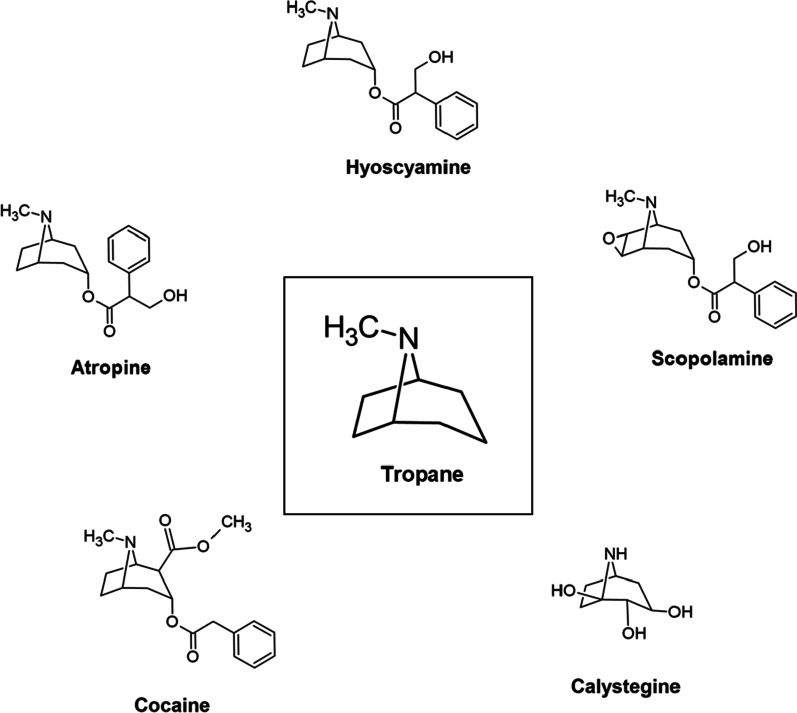
Structure of natural tropane alkaloids

Tropane is the condensation product of pyrrolidine precursor (ornithine) and piperidine ring with a common nitrogen and two carbon atoms [1]. In nature, TAs occur as ester formed by the combination of organic acids (tropic acid) and alcoholic base (tropanol). The structure of natural TAs is given in Fig. 2. We will not discuss about calystegines as despite of structural similarity, they are nortropanes due to the absence of the nitrogen associated methyl group. Also, they do not share the bioactivity of TAs [50] and behave as glycosidase inhibitors [51].

**Fig. 3:**
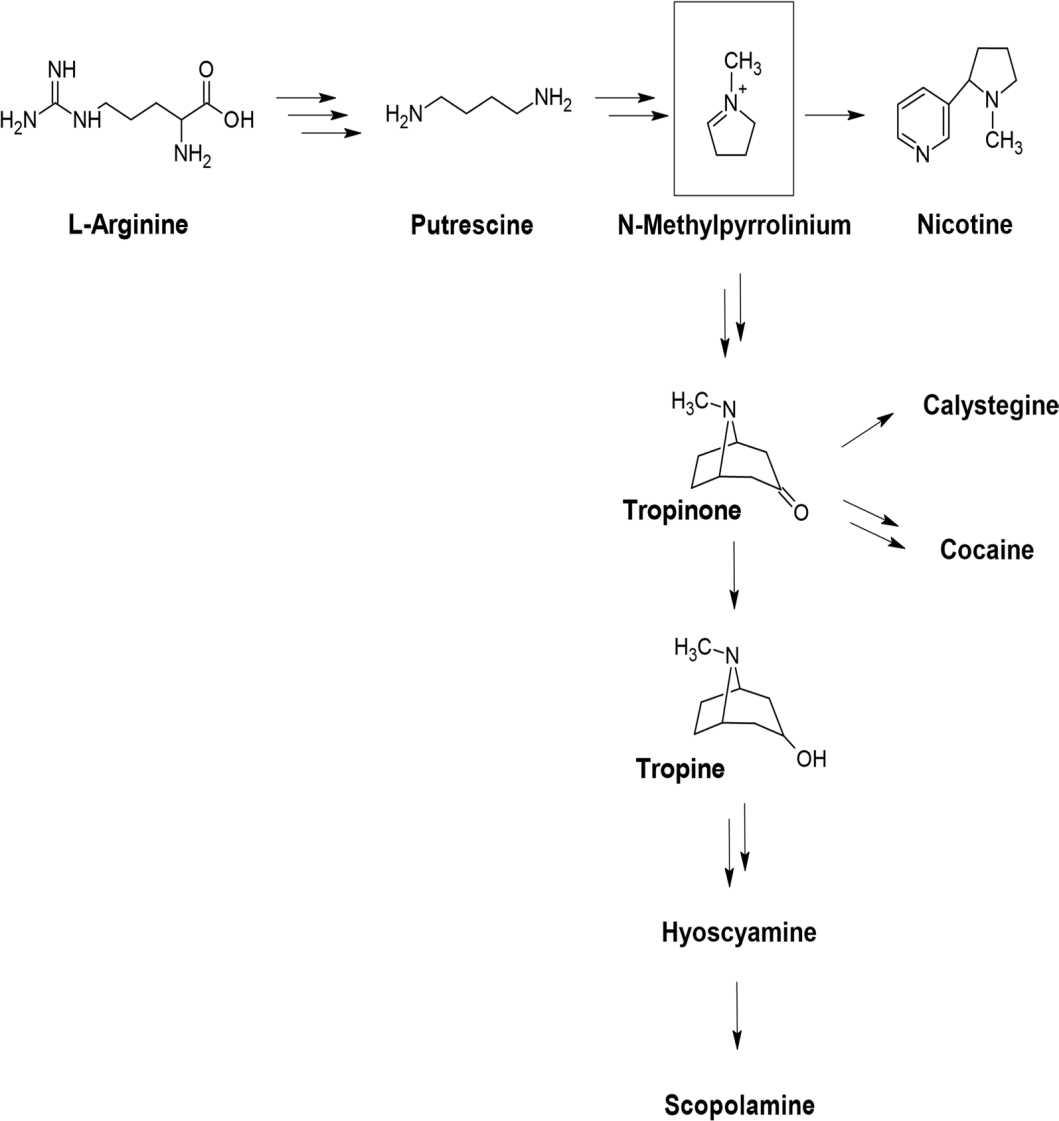
Biosynthetic pathway of tropane alkaloids

In plants, all the different types of TAs share a common biosynthetic pathway (Fig. 3) which starts with l-arginine (Arg). It undergoes a three-step decarboxylation and hydrolysis reactions to form Putrescine which gets methylated and undergo oxidative deamination to form *N*-methyl-Δ^1^-pyrrolinium, a branch point for the synthesis of TAs and nicotine.

### History

Atropine and scopolamine are found in several plants of Solanaceae family and have been used in ancient system of medicine for treating mental problems, skin diseases and tumors [28]. Atropine was the first TA to be isolated from the leaves and roots of *A. belladonna* by Mein in 1832 but he did not publish his results. A year later, Geiger and Hesse published the isolation of atropine from *Atropa belladonna* and *Hyoscyamus niger* [52]. Fifty years later, scopolamine was isolated from *Scopolia japonica* [53]. In 1864, Kraut and Lossen revealed the stereochemistry and cleavage products (tropic acid and tropine) of atropine and hyoscyamine [54]. Cocaine is considered as a ‘wonder medicine’ in the texts as it has local anaesthetic action and has role in treating postnatal depression and morphine overdose [37]. It was isolated by Gaedcke in the year 1855, from the leaves of *Erythroxylon coca* [55]. His research was later extended by Neimann [56] who not only improved the isolation method of cocaine but also elucidated its mode of action.

### Anticholinergic action

**Fig. 4:**
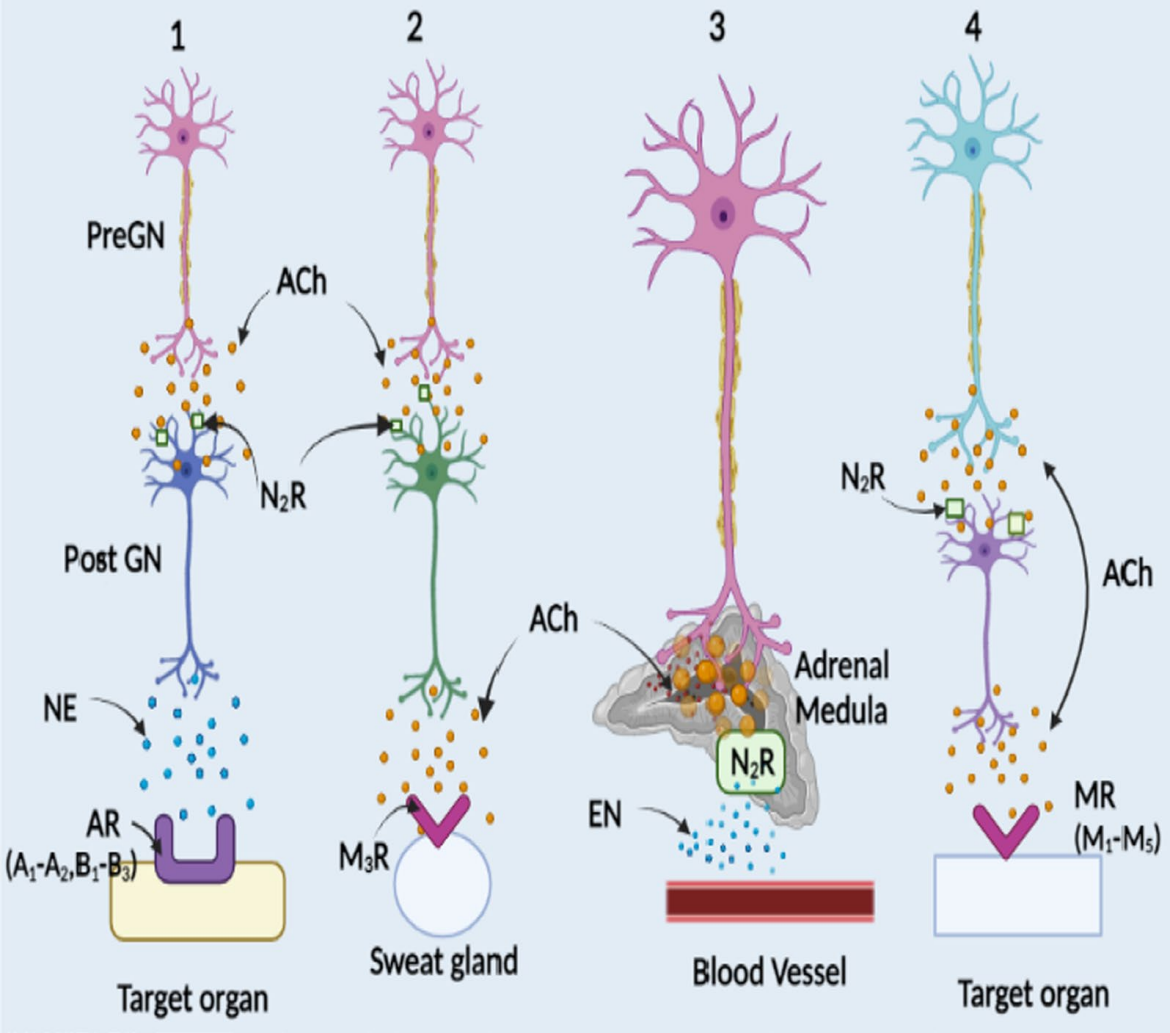
Autonomic nervous system and location of receptors. Nerves of autonomic nervous system (ANS) extend from central nervous system (CNS) to cardiac muscle, smooth muscle, organs and glands by preganglionic (Pre GN) and postganglionic (Post GN) neurons. PreGN releases acetylcholine (ACh) into the synaptic cleft and get bind to nicotine receptors (NR) in Post GN. Depolarization of membrane results in action potential which on arriving at the axon terminal releases neurotransmitter: ACh, norepinephrine (NE) or epinephrine (EN) in the synaptic cleft. The binding of neurotransmitter to the receptors: Androgenic (AR) or muscarinic (M) on the target organ results in excitation or inhibition. (1–3) Sympathetic system, (4) Parasympathetic system. These two systems differ in the type of neurotransmitter released, type of receptors and secondary messengers expressed [Prepared using Biorender.com]

TAs are anticholinergic in action i.e. they inhibit ACh mediated response by competitively binding to muscarinic and/or nicotinic receptors in the CNS and PNS (Fig. 1). There is almost no structural or physiological similarity between muscarinic and nicotinic receptors except for the fact that they both bind to ACh. Under the normal conditions, these receptors allow the binding of neurotransmitter ACh and help in signal transduction (Fig. 4).

Muscarinic receptors are G-protein-coupled receptors (GPCRs), more precisely rhodopsin-like (class A) receptors. Receptors M_1_, M_3_, and M_5_ activate phospholipase C by G*α*_q_ group whereas receptors M_2_ and M_4_ inhibit adenylate cyclase by G*α*_i_ group of G proteins [57]. The muscarinic acetylcholine receptors (mAChRs) are of five types (M_1_-M_5_) and dispersed all over the body [58]. In brain, they are predominantly located in the hippocampus, cerebral cortex, neostriatum and substantia nigra [59]. Receptor M_1_ is the most abundant receptor in the cerebral cortical region and is associated with memory and learning processes [58]. The non CNS locations of mAChRs are mainly, smooth muscle, cardiac muscle, ciliary muscle, salivary glands and sympathetic ganglia. Antagonism at M_1_ and M_2_ receptors have negative impact on memory and cognition [60]. In addition, pre-synaptic release of ACh is regulated by M_2_ receptor which is essential for the ACh homeostasis [61]. The regulation of other neurotransmitters (like dopamine) is altered by antagonism at M_4_ and M_5_ receptors. Interestingly, blocking of M_3_ receptor has apparently no effect on the cognition [60].

Neuronal nicotinic acetylcholine receptors (nAChRs) are cation-selective, ligand-gated ion channels, widely present in CNS and PNS. Seventeen different types of nAChRs have been identified including, ten α (α_1_–α_10_), four β (β_1_–β_4_), γ, δ, and ε subunits [62]. In brain, homomeric α_7_ nAChRs and heteromeric α_4_β_2_.^∗^ nAChRs are predominantly present [63, 64] and thought to have important role in the neurodegenerative diseases [65–67]. The nAChRs are also known to regulate dopamine release in the brain striatum. Cocaine reduces dopamine release in dopaminergic neurons by antagonizing α_4_β_2_-containing (α_4_β_2*_) nAChRs (IC_50_ 4–15 μM) [68, 69]. At high concentrations (40 µM), cocaine also inhibits voltage-gated Na channels (I_Na_) in dopanergic neurons [68]. Binding of nicotine and cocaine to nAChRs has rewarding effect on the brain. Thus, people are easily addicted to these compounds. Researchers confirmed the role of α_6_β_2*_ and α_4_β_2*_ in nicotine and α_6_β_2*_ nAChRs in cocaine addiction [70]

Atropine and scopolamine are non-selective competitive antagonist of muscarinic receptors. Atropine has the highest affinity for subtype M_1_, followed by M_2_ and M_3_ and weak affinity for M_4_ and M_5_ [71]. On the other hand, scopolamine has strong affinity for M_1_-M_4_ compared to M_5_ [72] while hyoscyamine binds to M_2_ only [73]. By stabilizing the receptors in inactive conformation TAs increase the intracellular levels of 3′,5′-cyclic adenosine monophosphate (cAMP) [74, 75]. Additionally, atropine inhibited phosphodiesterase (PDE4), causing increased heart contraction after β-adrenergic stimulus. This perspective might be helpful to elucidate cardiovascular adverse effects of atropine in part [76].

Both atropine and scopolamine displayed a high affinity towards mAChR in porcine brain (IC_50_ 4.7 nM and 2.2 nM, respectively). N-methylation of hyoscyamine and scopolamine increased the affinity (IC_50_ 0.1–0.3 nM) whereas removal of N-methyl group of atropine reduced the affinity considerably [77]. Scopolamine and hyoscyamine are known to bind to mAChRs exclusively. However, binding assay results clearly indicated that TAs can bind to both mAChR and nAChR. For example, atropine (mAChR: IC_50_ 4.7 nM; nAChR: IC_50_ 284 µM); scopolamine (mAChR: IC_50_ 2 nM; nAChR: IC_50_ 928 µM); cocaine (mAChR: IC_50_ 57 µM; nAChR: IC_50_ 371 µM) [77] bind to both the receptors but with different affinity. At higher concentration scopolamine also increased α7-nAChR expression [78]. The micromolar scale of activities might not be suitable for medical application of but certainly they enhance their toxicity under intoxication/overdose state, as with high concentration they can block both the receptors.

### Clinical pharmacology

For the pharmacological action, stereo-selectivity is the most important requirement since it effects the binding affinity of molecule to the receptor. It was observed that the *S*-(–)-hyoscyamine is around 30–300 times more potent than the *R*-( +)-isomer. However, being unstable, *S*-( −)-isomer quickly forms + hyoscyamine (+ atropine). As being a racemic mixture atropine is more stable hence is frequently used for clinical practice instead of its isomers [79].

TAs (atropine, scopolamine, and hyoscyamine) can be administered by multiple routes viz*.* oral, intravenous, intramuscular, endotracheal and transdermal [80]. Oral administration is more patient friendly, compared to transdermal application, and assures quick absorption and pharmacological action. Both atropine and scopolamine are easily absorbed from the gastrointestinal tract but the pharmacokinetics might vary in the patients [81]. However, the transdermal delivery is preferred for scopolamine as it reduces drastic drug variabilities and side effects, and more patient-friendly [82].

#### Atropine

Atropine or atropine sulfate is a US Food & Drug administration (FDA) approved drug. It is first line therapy for bradycardia, organophosphate poisoning and anti-sialagogue/anti-vagal action [83].

Depending on the route of administration the peak plasma levels vary from few min (Intramascular:13 min) to hours (Oral:1 h; Aerosol:1.5–4 h) [84]. Sex based variances in the pharmacokinetics of atropine was also observed as the AUC (Area under the curve) and C_max_ (peak plasma concentration) being 15% higher in females compared to the males. Additionally, female have shorter half-life of atropine (~ 20 min) than the males. Atropine binds to 14–22% plasma proteins [85]. Atropine is metabolised in the liver and almost 90% of the drug is cleared from the body through urine within 24 h [86]. In a clinical study, atropine (1 mg, intravenous) was administered to healthy volunteers and its plasma levels were analysed by radioimmunoassay. The maximum plasma atropine concentration correlated with the increased pulse rate between 12 and 16 min. However, no correlation between time of maximum response and atropine plasma concentration was perceived. Age is also an important factor in determining kinetics of atropine as both children and elderly are more sensitive to the atropine. In earlier times, rectal administration (0.01 mg/kg) was a preferred method of atropine delivery in children to slow down systemic absorption rate of atropine and this displayed better pre-operative sedation with lesser side effects [87]. Delayed time and lower peak plasma concentration of atropine was observed for rectal (15 min, 0.7 ng/ml) compared to intramuscular (5 min, 2.4 ng/ml) administration [88]. In conditions requiring high sedation, the use of 8 mg/kg ketamine, 0.5 mg/kg midazolam and 0.02 mg/kg atropine was found more effective in children [89]. The concentration of atropine should be decided very carefully as 0.3 mg/L of it is lethal in the peripheral blood [90].

Atropine metabolism significantly differs amid species on the basis of glucuronidation and N-demethylation reactions [91]. In rabbits, atropine was reported to be cleaved by serum carboxylesterase to tropic acid and tropine but such activity was not observed in serum from humans and monkeys [92]and the major metabolite (29%) was found to be tropine in human [93]. On the contrary, in another study, metabolism of radiolabelled atropine resulted in recovery of 57% radioactivity in urine as (+)-hyoscyamine followed by nortropine (24%), atropine oxide (15%), tropic acid (3%), and tropine (2%). However, no conjugated products of atropine with glucuronides or sulfates were observed [94].

#### Scopolamine

Scopolamine is an intravenous, oral, ophthalmic or topical drug having several uses including the prevention of motion sickness and postoperative nausea. It competitively blocks 5-hydroxytryptamine (HT_3_) receptors, with IC_50_ 2.09 µM [95]. Scopolamine is often administered to induce cognitive dysfunction in animal models. Apart from being a non-selective mAChR antagonist, scopolamine also inhibits G-protein coupled post-ganglionic muscarinic receptors. For this reason, it affects both CNS and PNS [96].

The half-life of scopolamine depends on the method of administration. For example, for oral (63.7 ± 1.3 min), intravenous (68.7 ± 1.0 min), intramuscular (69.1 ± 8.0 min) and subcutaneous administration (213 min) [97]. Similarly, the pharmacokinetics of scopolamine also depend considerably on the route of administration. The intravenous dose (0.5 mg) showed better results (C_max_ 5.00 ± 0.43 ng/ml, t_max_ 5.0 min, AUC 369.4 ± 2.2 ng min/ml) compared to the oral (C_max_ 0.54 ± 0.1 ng/ml, t_max_ 23.5 ± 8.2 min, AUC 50.8 ± 1.76 ng min/ml). However, rapid absorption (C_max_ 1.68 ± 0.23 ng/ml, t_max_ 2.2 ± 3 min, AUC 167 ± 20 ng min/ml) was observed after intranasal administration [97].

FDA has approved scopolamine transdermal therapy system patch (TTS-patch) [98] to overcome the dose-dependent adverse effects of the drug and to obtain therapeutic plasma concentrations over a longer period of time. The scopolamine in TTS-patch reaches protective levels in 6–8 h with optimum efficacy up to 72 h consequently a constant high plasm concentration (56–245 pg/ml) is reached. Whereas, oral or intravenous dose display quick effectiveness (0.5 h) but lasted for a short time (6 h). With a combined transdermal and oral administration, a peak at ∼0.37 ng/ml was reported after an hour [99]. Though scopolamine metabolizing enzymes are unknown, it is thought to be metabolised through oxidative methylation via cytochrome P_450_ (CYP_450_) and after Phase-II conjugation (gluronidation and sulphation) around 2.6% scopolamine is eliminate in the urine [97]. Metabolism of scopolamine is highly species-specific as metabolites p-hydroxy-, m-hydroxy- and p-hydroxy-m-methoxy-scopolamine are reported in rats while tropic acid was the major metabolite in rabbits and guinea pigs, but not in the mice [100].

In toxicity studies, oral dose of scopolamine displayed LD_50_ (Lethal dose for 50% deaths) of 1880 and 1270 mg/kg in mice and rats, respectively while the subcutaneous administration had much lower LD_50_ (1650 mg/kg and 296 mg/kg in mice and rats, respectively) [101].

#### Cocaine

After Phase 3 trials and Phase 1 pharmacokinetic study, FDA had approved Numbrino (cocaine hydrochloride) nasal spray as a topical solution for the nasal cavity mucus membrane [102]. Before the approval also the cocaine was in use as local anaesthetic agent, but later it was replaced by safer drugs.

After cocaine administration (intravenous/pulmonary), instantaneously high peak plasma levels are attained [103] and is cleared from the body within 4–6 h [104]. Pharmacokinetics of cocaine was studied in human subjects, after intravenous (32 mg, 1 ml/min) and intranasal (64 and 96 mg, inhaled with 5 cm straw/min) administration. Based on the one-compartment model data, intravenous administration displayed first-order elimination while intranasal exhibited both first-order absorption and elimination. The mean ± SEM half-life of cocaine through intravenous was 41.4 ± 8.2 min. AUC was significantly different in both type of administration [105]. In a similar study, oral (100 and 200 mg) and intravenous dose (40 mg) was given to volunteers and the cocaine metabolites were analysed by gas chromatography-mass spectrometry (GC–MS) [106] and identified as major (benzoylnorecgonine, ecgonine methyl ester) and minor (m- and p-hydroxybenzoylecgonine, m- and p-hydroxycocaine and norcocaine) metabolites [107]. Non-compartmental analysis and a two-factor model was used to evaluate the pharmacokinetic parameters. The bioavailability of cocaine was 0.32 ± 0.04, and 0.45 ± 0.06 after 100 and 200 mg oral dose, respectively. Both volume of distribution (V_d_) and clearance (C_L_) are found lowest for intravenous dose (V_d_: 1.3 L/kg; C_L_: 32.7 ml/min.kg) and highest for 100 mg oral dose (V_d_: 4.2 L/kg; C_L_: 116.2 ml/min.kg) compared to 200 mg oral (V_d_: 2.9 L/kg; C_L_: 87.5 ml/min.kg). The oral administration displayed a distinctive metabolic profile, with superior concentrations of major and minor metabolites as compared to the intravenous dose [106].

### Tropane alkaloid derivatives in therapeutics

**Fig. 5:**
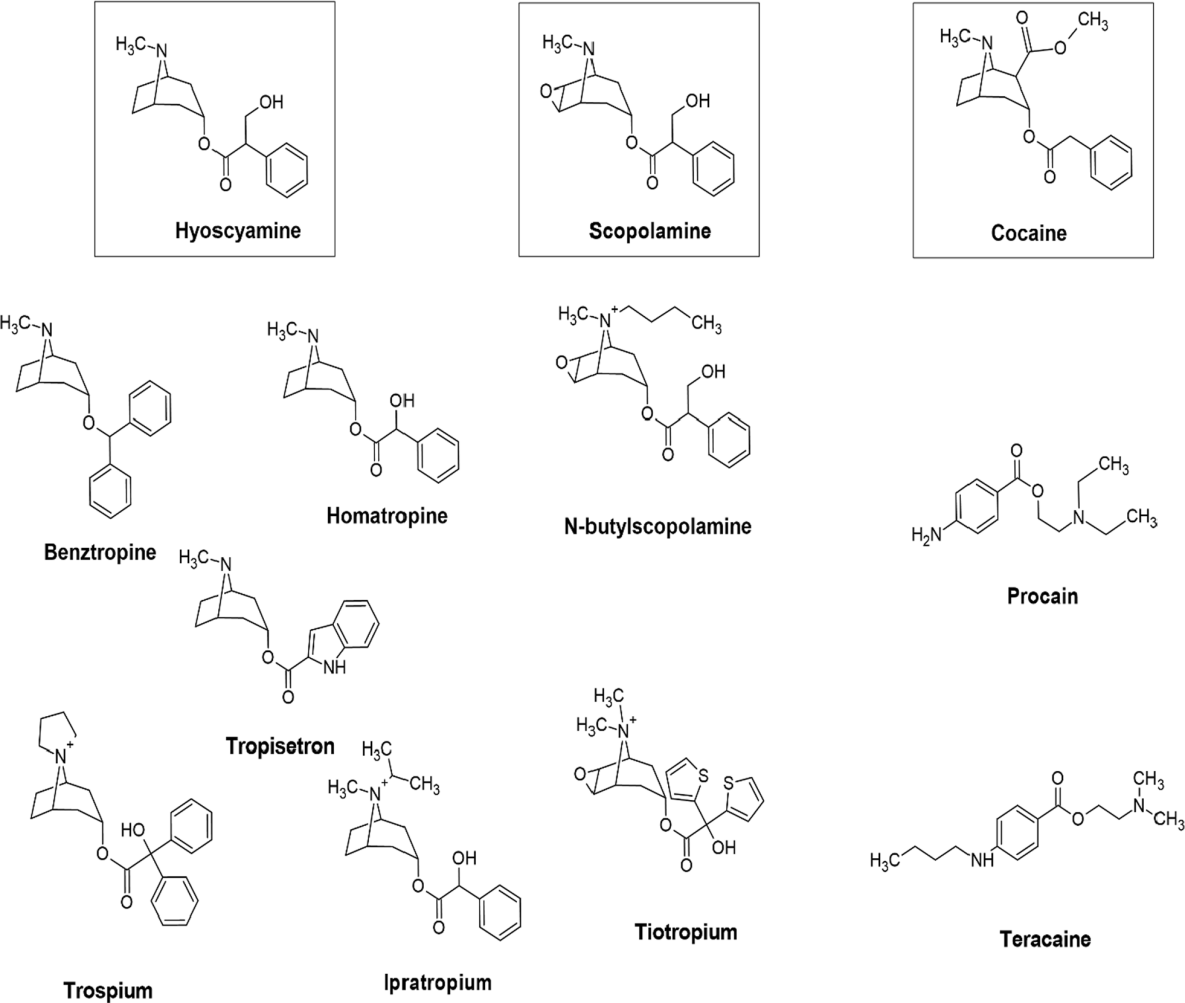
Pharmaceutically important tropane alkaloid derivatives

TAs serve as important lead molecules in the pharmacology. Various derivatives are synthesised and most of them have been approved by FDA. We will discuss about few important TA derivatives here (Fig. 5).

Scopolamine is prescribed to treat nausea and slobbering in the patients suffering from Alzheimer and Parkinson diseases (AD and PD) [108]. Motion sickness, postoperative nausea and vomiting can be treated by transdermal application of scopolamine [109, 110]. Additionally, being anti-muscarinic it produces antiemetic and sedative effect. Hyoscine is also used during endoscopic retrograde cholangiopancreatography (ERCP) for relaxing the smooth muscles [111]. Atropine is effective in treating nocturnal asthma by improving mucociliatory function of the lungs [112]. It also influences the vagal tone of diabetic patients [113] and controlled the tremors in monkey with PD [114].

USL Pharma developed benzotropine as a tropane-based dopamine inhibitor which was approved by FDA as adjunctive therapy for PD [115]. Benzotropine is a selective M_1_ mAChR antagonist which also inhibits dopamine uptake and decreases serotonin and norepinephrine inhibition. As a result, it is used in the symptomatic treatment for PD [116] and cocaine addiction [117]. Another tropane derivative, homatropine was launched as a mydriatic drug (dilate pupil) by Merck Darmstadt in 1883 [118]. Homatropine bind to muscarinic receptors in stomach and atria with similar affinity [119]. Homatropine is not FDA approved but a mixture of homatropine methylbromide and hydrocodone bitartrate has been approved by FDA as antitussive agent (inhibit cough) [120]. A quaternary ammonium derivative, trospium chloride, is a FDA approved drug, manufactured by Indevus Pharmaceutical Inc. [121]. Trospium is anti-spasmodic agent which is helpful in relaxing smooth muscles thus used to treat over active bladder [122]. Tropisetron is another tropane derivative which functions serotonin receptor antagonist. It is used as antiemetic and as analgesic in fibromyalgia [123].

N-Butylation of scopolamine yields N-butylscopolamine which is on the WHO “List of Essential Medicines”[124] and is used as anti-spasmodic and used to treat abdominal cramping, colic pain and bladder spasms [125]. Its use in animals has been approved by FDA [126] as Buscopan® (Boehringer Ingelheim's animal-health US Inc.).

Tiotropium is a FDA approved inhalation spray, Spiriva® [127]**.** It is a bronchodilator used in the management of chronic obstructive pulmonary disease (COPD). The drug bind well with M_1_-M_3_ receptors, more potent than ipratropium and is considered safe [128].

The first derivative of cocaine was procaine which was used in dentistry as anaesthetic and pain killer. Tetracaine [129] (STERI-UNIT®, FDA approved), a potent derivative of procaine is also used in local anaesthesia in minor surgeries [130]. Tetracaine mixed with oxymetazoline is better topical aesthetic for nasal procedures compared to cocaine [131]. It is used as local ophthalmic anaesthetic [132]. Another derivative, lidocaine (Xylocaine, FDA approved) is used on the skin to comfort itching and pain due to minor burns, eczema, insect bites. Lidocaine is a sodium channel blocker which ultimately decrease muscle contraction and result in vasodilation, hypotension and irregular heartbeat. Hence it is classified as class 1b anti-arrhythmic agent and is on the WHO List of Essential Medicines [8]. The mechanism of action (MOA) and the minimum effective dose of some important tropane alkaloids and derivatives have been summarized in Table 1.

**Table 1:** Important tropane alkaloids and derivatives used in therapeutics

Drug	MOA	Role	Minimum effective daily dose (mg) in adult	Refs.
Atropine products	Sodium channel antagonist; mAChR antagonist	Gastrointestinal antispasmodic	0.0582	[80, 133]
Benzotropine	M_1_ mAChR antagonist	Anti-parkinson	0.5	[133]
Cocaine hydrochloride	Adrenergic receptor agonist; Sodium channel antagonist	Topical anaesthetics	40.0	[134, 135]
Homatropine	mAChR antagonist	Gastrointestinal antispasmodic	6.0	[133]
Hyoscyamine	mAChR antagonist	Gastrointestinal antispasmodic	0.31	[133]
Lidocaine	Adrenergic receptor agonist; Sodium channel antagonist	Skin anaesthetics; Antiarrhythmic	< 300 (anaesthesia) 50 (IV bolus) followed by 1 mg/min continuous IV (arrhythmia)	[136] [137]
Scopolamine	mAChR antagonist	Antispasmodic	0.33 (Patch); 0.0195(Oral)	[133]
Tetracaine	Rynodine receptor; nAChR antagonist	Ophthalmic anaesthetics	0.5% (1–2 drops)	[132, 138, 139]
Tiotropium	M_1_-M_3_ mAChR receptors agonist	Bronchodilator	0.018 (Oral) 0.0025 (Inhalation)	[140, 141]
Triprolidine	Downregulation of histamine H1 receptor (H1R) by M_3_ mAChR	Antihistamine	10.0	[133, 142]
Tropisetron	Serotonin 5HT_3_-receptor antagonist	Antiemetic	2.0	[143]
Trospium	mAChR antagonist	Bladder antispasmodic	20.0	[133]

### Tropane alkaloid toxicity

The Solanaceae family plants (*Atropa*, *Datura*, *Hyoscyamus* species) which are rich in TAs are usually found growing in fields as weeds and pose the threat of accidental contamination in food and feed during harvesting or processing. The highest TAs levels have been reported in herbal teas which are found to be mostly contaminated with leaves and berries of *A. belladonna* [144] with extremely high atropine (> 30 mg/g) affirmed in one of the cases [145]. The unprocessed cereals (like buckwheat, maize, millet, sorghum, wheat) and the cereal-based foods prepared from them are often adulterated (~ 7%–27%) with TAs (atropine, scopolamine) [144]. Surprisingly, presence of TAs (11.5 µg/kg of atropine; 2.8 µg/kg of scopolamine) was also detected in baby food samples which was well above the EU regulatory limit (1 µg/kg for each alkaloid) [146]. Seeds of *D. stramonium* are the most common contaminants in the food, followed by *H. niger* seeds, and berries of *A. belladonna* [46, 147, 148]. They are the main culprits of numerous cases of food poisoning worldwide [45, 149–151]. TA toxicity in livestock and other grazing animals is exceptional as the plants containing TAs are generally inedible and animals avoid feeding on them. However, consuming feed contaminated with TAs (like millet, corn etc.) can lead to toxicity [148].

TAs poisoning is common in children following consumption of attractive and colourful berries of Solanaceae family plants (*A. belladonna*, *H. niger*) [152] The symptoms of TA toxicity appear within 2 h of oral intake [153]. The classic signature of TAs poisoning can be identified from the mnemonic “red as a beet (flushing), dry as a bone (dry mouth), blind as a bat (mydriasis), mad as a hatter (mental confusion), hot as a hare (fever), full as a flask (urinary retention) [154]. Absence of sweating makes anticholinergic toxicity different from sympathomimetic toxicity [155]. The lasting effects of TAs on CNS is much longer (over 8 h) as compared to the cardiovascular system [156].

The treatment of TA poisoning including gastric emptying, use of activated charcoal (0.5 to 1 g/kg in children or 25 to 100 g in adults) to absorb the drug and benzodiazepines for managing agitation [157, 158]. Physostigmine (an AChE inhibitor) is recommended in the case when both PNS and CNS are affected by anticholinergic poisoning [159, 160]. In such cases, intravenous dose of physostigmine (0.02 mg/kg for children and 0.5 to 2 mg/kg for adults) is recommended [159]. Physostigmine is helpful in restoring the level of consciousness to its baseline [157] which is different from sedative action of benzodiazepines.

### Anticholinergic burden

The continuous use of medications with anticholinergic action (MACs) for a long time might cause cognitive decline, which is linked to the cholinergic changes [161] and increased the deposition of amyloid-beta (Aβ) peptide in several regions of brain including amygdala, cortex and hippocampus [162] A study reported increased frequency of neurofibrillary tangles (NFTs) in PD patients treated with MACs for a long time compared to short-term or no treatment [163]. However, these results were not proven in a related study [164]. Various contradictory assumptions are available in the literature regarding the reversibility of cognitive effects after discontinuation of MACs. As per some studies, withdrawal of MACs resulted in the improvement in cognitive function [165, 166] while such changes were not reported by another study [133, 167]. Additionally, discontinuation of treatment might worsen the disease state in some cases of psychotic or bipolar disorders [168].

The collective effect of anticholinergic medicines, also known as anticholinergic burden (AB), has been linked with detrimental effect on mental health of elderly which include cognitive debility [169–171], delirium [172, 173] and falls [174] especially in elderly. Recently, a study conducted by Reinold et. al. (2021) included a very large population size (~ 16 million) to accurately evaluate the predominance of AB on the basis of gender and age. A list of MACs, based on the German health care system, were evaluated in this study to assess AB using the Acetylcholinergic Cognitive Burden (ACB) scale. Total ACB score ≥ 1 signifies AB while ACB ≥ 3 suggests clinically substantial ACB [175] Interestingly, the highest share (39–86%) of total cumulated AB came from the MAC prescribed by general physicians. The most frequently prescribed MACs were for psychiatry (42.8%) and urinary diseases (40.2%) [176]. In addition, the study results revealed that AB is prevalent not only in younger population [177] but almost in all age groups [176]. Moreover, the clinically relevant AB was found higher in female compared to male population [177] and AB was highest in the elderly (43.2%), followed somewhat equally by the adults (25.8%) and children (20.7%).

The ACB score of various MACs including TA was also evaluated. Interestingly, least ACB scores (ACB score 1 and 2 = 0; ACB score 3 = 0.6%) are observed for TAs in the studied population, as compared to the other MACs. While, the maximum score was observed for the antidepressants (ACB = 1: 8.8%; ACB = 2: 22%; ACB = 3: 45.3%). The contribution of TAs to cumulative ACB in males (M) and females (F) was also calculated in various age groups: age ≤ 19 (2.0% M; 1.9% F), age 20–34 (0.1% M; 0.1% F), for all other age groups (age 35–49, 50–64, 65–79 and 80–94 and ≥ 95) cumulative ACB was 0.1% M and 0.0% F [177] This study negates the role of TAs in contributing to AB. Unfortunately, in the absence of additional such population based studies on TAs it is not possible to compare these results and come out with a conclusion.

## Conclusion

TAs represent a big cluster of secondary metabolites, predominantly present in the Solanaceae family. Naturally occurring TAs include atropine, hyoscyamine, scopolamine, cocaine and calystegine. The concentration of TAs in the plant is dependent on many biotic and abiotic factors. Therefore, alternative production system like genetically engineered plants, climate independent production using plant and microbial cultures are more desirable than the field grown system. Accidental contamination of cereals by Solanaceae family plants (seeds/berries) is common during harvesting and results in TA toxicity, both in food and feed. Thus, to avoid cross-contamination, good agricultural and collection practices emphasized by the World health organisation (WHO) should be strictly followed. Additionally, deoxyribonucleic acid (DNA) barcoding is a popular tool to detect and eliminate the contaminants.

TAs are anticholinergic in action and block ACh transmission by binding to muscarinic and/or nicotinic receptors. Except for calystegine, all others can cross BBB and exert effect on CNS. They are clinically used to minimize salivation and respiratory secretions, treat overactive bladder, and as antipsychotics. The anticholinergic activity affects both PNS (dry mouth, constipation, blurry vision, tachycardia, urinary retention) and CNS (drowsiness, confusion, vertigo). However, in spite of the side effects TA treatment work wonders in the clinically critical situations such as death rattle and organosulphate poisoning cases. Additionally, the derivatives of TAs with better efficacy and lesser side effects are already in therapeutic use after FDA approval.

Being anticholinergic in action, TA medication are under the suspicion of causing dementia and cognitive decline like other MACs. However, a recent study [177] was conducted on over 16 million populations to describe the incidence and classes of medicines contributing to AB. Surprisingly, TA medication was at the bottom of the MAC list with a nominal ABC score, suggesting an insignificant contribution to AB. Yet, as this study lack evidence on medications used during hospitalization and over-the-counter (OTC) medicines, more studies are required to access the participation of TAs in AB.
